# The microRNA-455 Null Mouse Has Memory Deficit and Increased Anxiety, Targeting Key Genes Involved in Alzheimer’s Disease

**DOI:** 10.3390/ijms23010554

**Published:** 2022-01-05

**Authors:** Tracey E. Swingler, Lingzi Niu, Matthew G. Pontifex, David Vauzour, Ian M. Clark

**Affiliations:** 1 School of Biological Sciences, University of East Anglia, Norwich NR4 7TJ, UK; t.swingler@uea.ac.uk (T.E.S.); l.niu@uea.ac.uk (L.N.); 2 Norwich Medical School, University of East Anglia, Norwich NR4 7TJ, UK; m.pontifex@uea.ac.uk

**Keywords:** Alzheimer’s disease, microRNA, miR-455, knockout, *APP*, *TAU*, *BACE1*, novel object recognition, memory, anxiety

## Abstract

The complete molecular mechanisms underlying the pathophysiology of Alzheimer’s disease (AD) remain to be elucidated. Recently, microRNA-455-3p has been identified as a circulating biomarker of early AD, with increased expression in post-mortem brain tissue of AD patients. MicroRNA-455-3p also directly targets and down-regulates APP, with the overexpression of miR-455-3p suppressing its toxic effects. Here, we show that miR-455-3p expression decreases with age in the brains of wild-type mice. We generated a miR-455 null mouse utilising CRISPR-Cas9 to explore its function further. Loss of miR-455 resulted in increased weight gain, potentially indicative of metabolic disturbances. Furthermore, performance on the novel object recognition task diminished significantly in miR-455 null mice (*p* = 0.004), indicating deficits in recognition memory. A slight increase in anxiety was also captured on the open field test. *BACE1* and *TAU* were identified as new direct targets for miR-455-3p, with overexpression of miR-455-3p leading to a reduction in the expression of *APP*, *BACE1* and *TAU* in neuroblastoma cells. In the hippocampus of miR-455 null mice at 14 months of age, the levels of protein for APP, BACE1 and TAU were all increased. Such findings reinforce the involvement of miR-455 in AD progression and demonstrate its action on cognitive performance.

## 1. Introduction

There are approximately 50 million people worldwide currently living with dementia, and this figure is predicted to rise considerably over the next three decades [1,2]. Alzheimer’s disease (AD) is the most common form of dementia accounting for approximately two thirds of dementia cases [3]. The progressive and debilitating nature of the disease make it a major cause of disability and mortality in later life, with no disease modifying treatment currently available [4]. The classic hallmarks of AD consist of cognitive decline and the presence of amyloid plaques and neurofibrillary tangles (NFT) [5]. According to the amyloid hypothesis [6], pathological conditions lead to altered/increased metabolism of the amyloid precursor protein (APP), under the actions of the β-secretase, BACE1 and γ-secretases, presenilin 1 and 2 (PSEN1 and PSEN2). APP cleavage leads to the production and aggregation of the amyloid β peptide (Aβ) and the formation of amyloid plaques, resulting in neurotoxicity and eventual death of the neuron [7,8]. On the other hand, the TAU (microtubule-associated protein tau) hypothesis [9] emphasises the importance of aberrant TAU phosphorylation and subsequent NFT formation, which undermines microtubule integrity resulting in structural and transport deficits in the neurons [10,11]. Further processes such as neuroinflammation have also been proposed to explain the mechanistic basis of the disease, with proinflammatory cytokines elevated in the brains and serum of AD patients [12,13]. Mitochondrial dysfunction and oxidative stress (increased production of reactive oxygen species) are also seen [14]. AD is likely attributable to a combination of processes, adding to its complexity, and as a result, the overall molecular pathogenesis remains poorly understood.

Small non-coding RNAs known as microRNAs (miRNAs) are important regulators of gene expression in human cells [15]. MicroRNAs are transcribed as primary transcripts (pri-miRNA) and processed to short stem-loop structures (pre-miRNA) in the nucleus. The pre-miRNA is then processed by the ribonuclease, DICER, forming two complementary short RNA molecules one of which (the guide strand) is integrated into the RNA-induced silencing complex (RISC), the other of which (the passenger strand) is degraded. After integration into RISC, miRNAs base pair with their complementary mRNA targets, usually in the 3′UTR [16] to degrade mRNA or repress translation. A number of miRNAs have been shown to have roles in regulating AD associated proteins, targeting processes that contribute to β-amyloid production (e.g., *BACE1* and *APP*), TAU phosphorylation and mitochondrial dysfunction (reviewed in [17,18]). MicroRNAs and their target genes act in regulatory networks which are disrupted in disease [19]. Key miRNAs may act as biomarkers of pathology or even be targets for therapeutic intervention [18].

MicroRNA-455 is genomically located in an intron of the collagen type XXVII alpha 1 chain gene (*COL27A1)* and has previously been implicated in cartilage biology [20], with roles in the TGFβ signalling pathway [20,21]. A number of studies have shown miR-455 to be dysregulated in a variety of pathologies including cancers and cardiovascular disease [22]. Recently, microRNA-455-3p was identified as a circulating biomarker in early AD [23] with levels of miR-455-3p upregulated in patients with mild cognitive impairment (MCI) and AD. Furthermore, miR-455-3p expression is increased in the post-mortem brain tissue and cells derived from AD patients when compared to healthy controls, [24]. MicroRNA-455-3p has also been shown to directly target and down-regulate APP, with the overexpression of miR-455 suppressing APP toxicity [25] providing a mechanistic explanation for its increased expression in AD.

In order to determine the function of miR-455 in vivo, we utilised a miR-455 null mouse, constructed using CrispR/Cas9. Behavioural tests of cognition and in vitro molecular analyses were employed to further delineate miR-455 function within the brain.

## 2. Results

### 2.1. MicroRNA-455-3p Expression Decreases with Age and Is Absent in the Null Mouse Model

**Figure 1:**
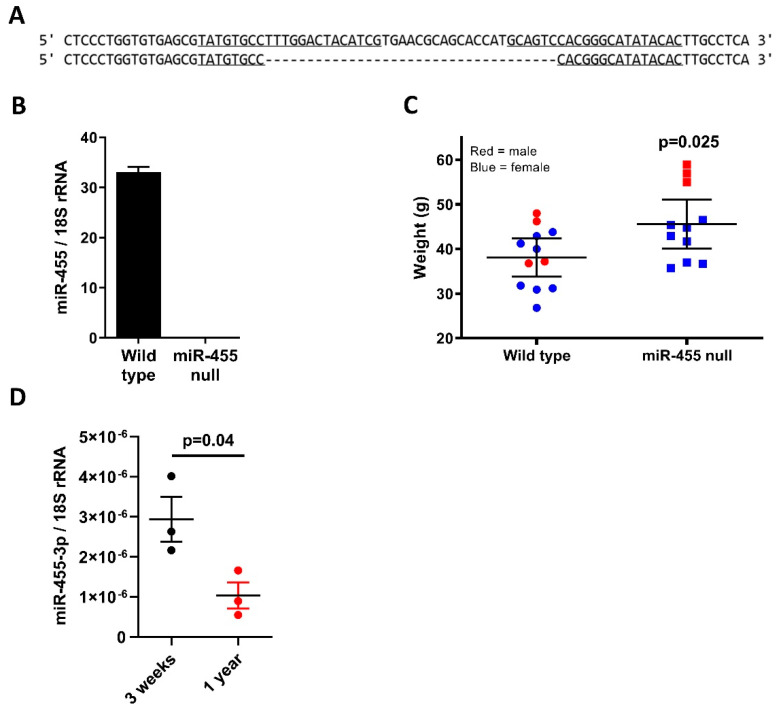
The miR-455 null mouse and the expression of miR-455-3p across age. (**A**) Deletion of miR-455 in mice. Sequencing of miR-455 null founder mouse shows a 35-base deletion, deleting both miR-455-5p and miR-455-3p mature sequences, as well as the intervening hairpin sequence. (**B**) Expression of miR-455-3p in 3-month-old mouse brain tissue. RNA was extracted from brain tissue and qRT-PCR performed; the miR-455 null mouse shows complete loss of miR-455-3p expression. (**C**) MiR-455 null mice show increased weight gain with age. Mice were weighed each month and showed that at 14 months of age, miR-455 null mice (*n* = 13) were significantly heavier than wild-type litter mates (*n* = 11). (**D**) MiR-455 expression decreases with age in wild-type mice. RNA was extracted from brain tissue at 3 weeks and 12 months of age and qRT-PCR performed (*n* = 3). Mean ± SEM.

The miR-455 null mouse was made by the Genome Editing Unit, University of Manchester, UK. Microinjection of one-day single cell C57/BL6 mouse embryos used in vitro transcribed sgRNA and recombinant Cas9 protein (https://sites.manchester.ac.uk/genome-editing-unit/publications/, accessed on 15 October 2021). Sequencing of genomic DNA from founder mice revealed a number of deletions within the targeted pre-miR-455 locus. One of these was identified as a 35-base deletion, removing part of both miR-455-5p and miR-455-3p mature sequences, as well as the intervening hairpin sequence (Figure 1A). This mouse was bred with a wild-type C57/BL6 and heterozygote offspring bred together to give homozygous null mice; these were maintained alongside wild-type littermates as parallel colonies. Deletion was confirmed by sequencing of genomic DNA.

Expression of miR-455 was absent in all tissues tested, with Figure 1B showing expression in the brain at 3 months of age. At 14 months of age, miR-455 null mice were significantly larger than the wild-type comparators (Figure 1C). Expression of miR-455-3p in the brain decreased across 3 weeks to 12 months of age in wild-type mice (*p* = 0.04, Figure 1D).

### 2.2. Recognition Memory Is Impaired in miR-455 Null Mice

**Figure 2:**
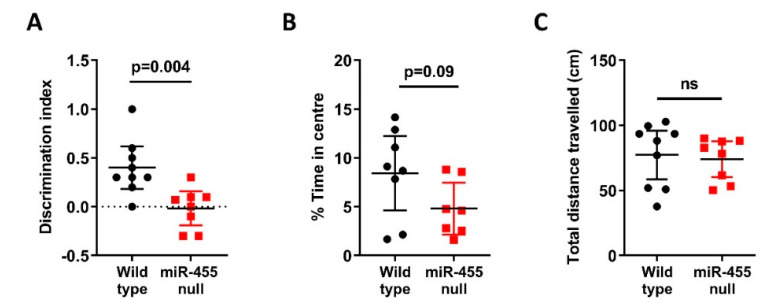
Behavioural studies comparing the miR-455 null mouse vs. wild-type. (**A**) 14-month-old miR-455 null (*n* = 9) mice and wild type mice (*n* = 8) were tested for recognition memory using the novel object recognition test (NOR); miR-455 null mice have significant recognition memory deficit as indicated by a decreased discrimination index. (**B**) Anxiety behaviour was tested using the open field test. MiR-455 null mice display an increase in anxiety with (**C**) no difference in overall locomotion. Bars show the mean ± SEM.

We compared wild-type with miR-455 null mice at 14 months of age in both novel object recognition and the open field test. In object recognition, miR-455 null mice showed significantly impaired recognition memory compared to wild-type controls (*p* = 0.004, Figure 2A). In the open field test, miR-455 null mice displayed a slight increase in anxiety overall spending less time in the centre of the arena (*p* = 0.09, Figure 2B), with no alteration in motor function (Figure 2C).

### 2.3. BACE1 and TAU Are Direct Targets of miR-455-3p

**Figure 3:**
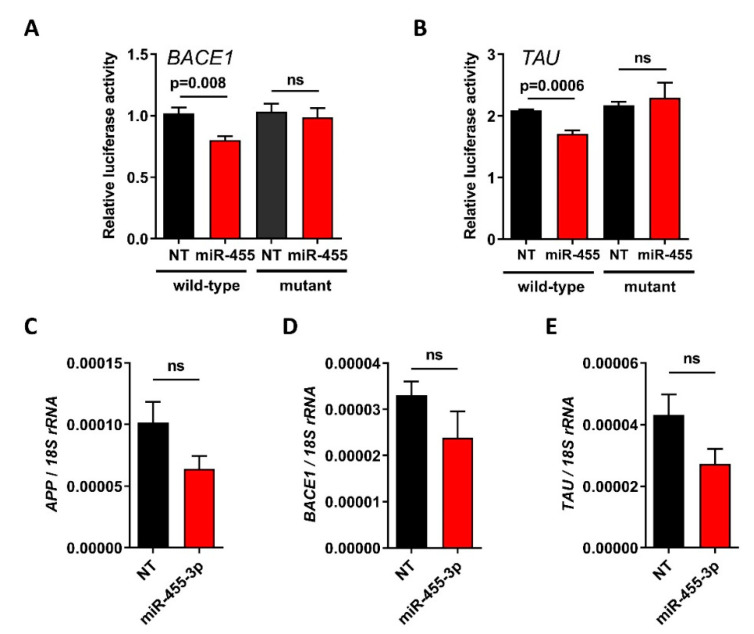
miR-455-3p directly targets *BACE1* and *TAU*. Cells (DF1 fibroblasts) were transfected with the 3′-untranslated region of *BACE1* (**A**), *TAU* (**B**) or the corresponding seed site mutants, cloned into pmiR-GLO ± non-targeting control (NT) or miR-455-3p mimic (miR-455) and incubated for 24 h. Relative light units were normalized to Renilla activity. Bars show the mean ± SEM; *n* = 4. SH SY5Y neuroblastoma cells were transfected with miR-455-3p mimic or non-targeting control (NT) for 48 h prior to measurement of *APP* (**C**), *BACE1* (**D**) and *TAU* (**E**) by qRT-PCR. Bars show the mean ± SEM, *n* = 3.

*APP* has previously been identified as a direct target for miR-455-3p [25]. Here, we also identified *BACE1* and *TAU* as other targets. Expression of luciferase controlled by the 3′UTR of either the *BACE1 or TAU* genes shows that miR-455-3p reduces this expression and that this is rescued by mutation of the miR-455-3p seed site in each UTR (Figure 3A,B). Expression of *APP*, *BACE1* and *TAU* were all decreased after overexpression of miR-455-3p in SH SY5Y neuroblastoma cells (Figure 3C,D).

### 2.4. The Levels of APP, BACE1 and TAU Proteins Are Increased in miR-455 Null Mouse Hippocampus

**Figure 4:**
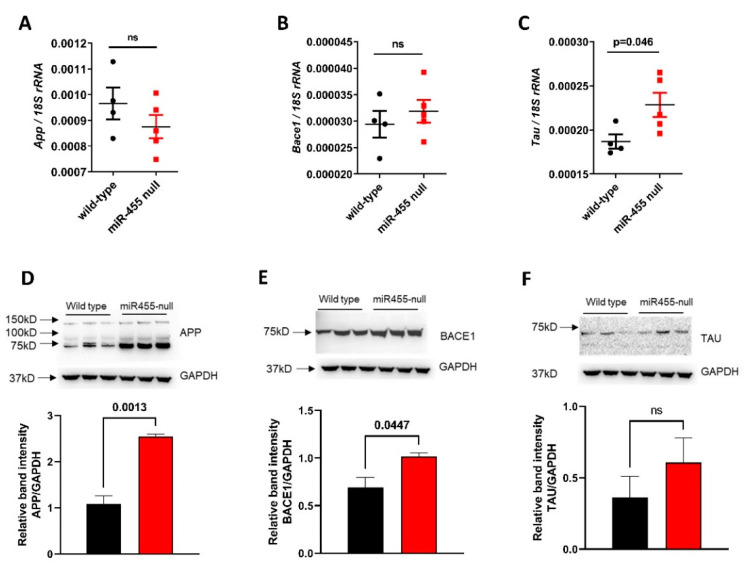
Expression of *App*, *Bace1* and *Tau* in the mouse hippocampus. RNA and proteins were extracted from the hippocampus of wild-type vs. miR-455 null mice at 14 months of age. Levels of *App*, *Bace1* and *Tau* were measured by qRT-PCR (**A**–**C**) and Western blot and densitometric analyses (**D**–**F**). Results are presented as mean ± SEM, *n* = 4 wild type and *n* = 5 miR-455 null for RNA levels and *n* = 3 for both wild type and miR-455 null for protein levels.

Both RNA and proteins were extracted from the hippocampus of wild-type vs. miR-455 null mice at 14 months of age. The level of both *App* and *Bace1* gene expression were not significantly different between groups (Figure 4A,B), although *Tau* expression was significantly increased in the miR-455 null mice (*p* = 0.046, Figure 4C). However, Western blot showed a significant increase in APP (*p* = 0.0013) and BACE1 (*p* = 0.0447) protein levels in miR-455 null mice compared to wild-type (Figure 4D,E). Although it did not reach significance, protein levels of TAU were higher in miR-455 null vs. wild type mice (Figure 4F).

## 3. Discussion

MicroRNA-455 has been described as a circulating biomarker of AD and is increased in the brains of AD patients [22,23,24]. Additionally, miR-455-3p has known roles in APP processing and abrogates the impact of mutant APP in vitro [25]. After revealing a decrease of miR-455-3p expression in ageing wild type mice, CrispR-Cas9 microinjection was used to create a miR-455 null mouse in order to determine its function in vivo. Behavioural assessment revealed deficits in recognition memory and increased anxiety in null mice. *BACE1* and *TAU* genes were shown to be direct targets for miR-455-3p. The use of homologous recombination was precluded because of the proximity of miR-455 to the downstream exon of the *COL27A1* gene. The deletion of 35 bases across the 5p and 3p sequences within the pre-miRNA hairpin was sufficient to prevent processing and no mature miR-455 was detected. A second deletion in founder mice was also bred forwards and no difference in phenotype of these mice has been detected (data not shown).

Expression of miR-455-3p decreased with age in the mouse brain across 12 months (Figure 1D). The expression of all microRNAs in mouse brain with ageing has previously been examined using RNA-Seq analysis [26,27], however n numbers were small and the read number of miR-455 using this technique was too small for analyses. The expression of a number of microRNAs both increase and decrease in the brain significantly across age. Roles for miRNAs in the brain are diverse, including modulation of synaptic plasticity, cognition, inflammation, neuroprotection, lipid metabolism and mitochondrial function [28,29].

MicroRNA-455 null mice were significantly heavier than their wild-type counterparts at 14 months of age (Figure 1C) and this divergence starts around 6 months of age (Supplementary Figure S1). Although the role of obesity as a risk factor for AD remains uncertain [30,31] alterations in energy metabolism are now considered a prerequisite in AD progression [32,33,34,35].

Loss of miR-455 influenced recognition memory with null mice performing significantly worse on the novel object recognition task compared to the wild-type controls (Figure 2A). A slight increase in anxiety, was also noted Figure 2B). Correlation analyses of NOR discrimination index with open field percentage time in centre or travelled distance showed no significant interaction indicating that anxiety did not impact on the NOR output (see Supplementary Figure S2). Several microRNAs implicated in AD have also been shown to impact these outcomes, including miR-9 [36], miR-124 [37], miR-101 [38], miR-153 [39], miR-132/212 [40] and miR-181a [41,42].

MicroRNA-455 has been shown to directly target and downregulate APP [25], suppressing its toxic effects. We extended this to demonstrate that both human *BACE1* and *TAU* are also direct targets for miR-455-3p (Figure 3A,B) with *APP*, *BACE1* and *TAU* expression suppressed by the overexpression of miR-455-3p (Figure 3C–E). In the miR-455 null mouse hippocampus, the protein levels of APP, BACE1 and TAU are all increased, though for APP and BACE1, this does not correlate with mRNA levels. In part this may be due to a small n number, but post-transcriptional mechanisms may also contribute. This clearly has potential to add to the pathology of AD via the amyloid or *Tau* axis. Although *APP*, *BACE1* and *TAU* have been implicated in impaired recognition memory [43,44,45,46] we have not demonstrated that this is the mechanism of action of miR-455.

In addition to our current findings, we have preliminary evidence to suggest miR-455 may also target components of the core clock machinery and likely impacts the upon circadian rhythm (Swingler et al., unpublished observations). This may provide another link to AD [47].

The main limitations to the current study are the future need to compare the miR-455 null mouse with a transgenic mouse overexpressing miR-455-3p. A greater suite of behavioural studies across age would identify the age of onset for the phenotype and enable targeted molecular studies to address mechanism further.

Altogether, the findings here suggest that miR-455-3p is neuroprotective, with loss of miR-455 across age leading to increased AD related gene expression and subsequent cognitive deficits. However, the knowledge that miR-455-3p [22] is increased in AD complicates this narrative. We hypothesize that the AD associated increase may represent a futile attempt to control pathology and is ultimately overwhelmed. Increasing research is warranted to further detail the interaction of miR-455 with brain physiology and pathology.

## 4. Materials and Methods

### 4.1. MiR-455 Null Mouse

MiR-455 mice were made using CRISR-Cas9 by the Transgenic Unit, University of Manchester (https://sites.manchester.ac.uk/genome-editing-unit/, accessed 15 October 2021). Mice were maintained in a controlled environment under the Home Office Code of Practice (21 +/− 2 °C, humidity 55 +/− 10%, 12-h light/dark cycles (lights on at 07:00 h and off at 19:00 h), HEPA filtered air) and fed ad libitum on a standard chow diet (RM3-P; Special Diet Services, Essex, UK) for the duration of the experiments.

### 4.2. Behavioural Assessment

Behavioural tests were performed as previously described [48,49] on mice aged to 14 months old. Objects and arenas were cleaned with 70% (*v/v*) ethanol in between each trial. To evaluate anxiety behaviour and locomotion open field test was performed. Briefly, mice were placed in a house built 50 cm × 50 cm × 50 cm apparatus illuminated with low lux (100 lux) lighting and could move freely. Recognition memory was assessed using novel object recognition task (NOR) [50]. The open field test (Day 1) was used as the acclimatisation phase for the NOR experiment. On day 2, mice were placed into the same arena containing two identical objects and allowed to explore for 15 min. The mice were returned to their cages for a period of 1 h. Animals were again placed into their respective arenas for a final time, the arena contained one original object and one novel object, and the mouse was free to explore either for a 10-min period. Mice were included in the analysis that explored objects for a period of 10 secs or greater. Videos were analysed for the full the 10-min period and time spent with each object was determined. Discrimination index (DI) was calculated using the following formula DI = (TN − TF)/(TN + TF) where TN is time exploring the novel object and TF is time spent exploring the familiar object.

### 4.3. Overexpression of miR-455-3p

SH SY5Y neuroblastoma cells were plated in 96-well plate wells at a density of 7 × 10^4^ cells/mL in 100 µL and grown to 80–90% confluence. MicroRNA-455-3p mimic (50 nM) or non-targeting control (50 nM) were transfected in using Lipofectamine 3000 (Thermo Fisher Scientific, Paisley, UK), according to manufacturer’s instructions for 48 h. Total RNA was extracted using Trizol (ThermoFisher Scientific, Paisley, UK) according to the manufacturer’s instructions).

### 4.4. RNA and Protein Extraction from Mouse Brains

Mice were euthanized and the brains were immediately removed and dissected for the cerebral cortex, and hippocampus. Brain tissue was snap frozen and ground in liquid nitrogen. Total RNA was extracted from 20 mg tissue using Trizol (ThermoFisher Scientific, Paisley, UK) according to the manufacturer’s instructions. Protein was extracted using NE-PER reagents (ThermoFisher Scientific, Paisley, UK) according to the manufacturer’s instructions.

### 4.5. Quantitative Real-Time PCR

Complementary DNA was synthesized from 300 ng RNA using SuperScript II reverse transcriptase (Invitrogen, Paisley, UK) and either random hexamers or miRNA-specific primers (Applied Biosystems, Paisley, UK) according to the manufacturer’s instructions. 18S rRNA was used as the housekeeping gene. Complementary DNA was stored at −20 °C. The relative quantitation of gene expression was performed using an ABI Prism 7700 Sequence Detection System (Applied Biosystems, Paisley, UK), following the manufacturer’s protocol.

### 4.6. Luciferase Assay

The 3′UTR of mRNAs containing the predicted binding site of miR-455-3p were subcloned into pmirGLO (Promega, Chilworth, UK), using QuikChange II XL site-directed mutagenesis kit (Agilent, Stockport, UK) to introduce mutations. Constructs were sequence verified (Source Bioscience, Nottingham, UK). DF1 fibroblast cells were seeded into 96-well plate wells at 5 × 10^4^ cells/mL in 100 µL medium overnight and transiently transfected with 100 ng reporter plasmid, 30nM miR-455-3p mimic (Qiagen, Manchester, UK) or non-targeting control (AllStars, Qiagen, Manchester, UK) using Lipofectamine 3000 (Thermo Fisher Scientific, Paisley, UK), according to manufacturer’s instructions for 24 h. Cell lysates were assayed for luciferase using the Dual Luciferase Reporter Assay Kit (Promega, Chilworth, UK), read with an EnVision 2103 Multilabel plate reader (Perkin Elmer, Beaconsfield, UK). Relative luciferase activity was the ratio of firefly luciferase to Renilla luciferase activity

### 4.7. Western Blot

Samples were separated on reducing SDS-PAGE, transferred to PVDF membrane and probed overnight at 4 °C. Antibodies against APP (A87840), BACE1 (A80396) and TAU (A98434) were all from antibodies.com (Cambridge, UK). The antibody against GAPDH (#2118) was from Cell Signalling Technology (London, UK). All antibodies were used at recommended concentrations and were detected using HRP-conjugated secondary antibodies (DAKO, Stockport, UK), visualised using Pierce™ ECL Western Blotting Substrate (ThermoFisher Scientific, Paisley, UK), and imaged by ChemiDoc™ MP Imaging System (BioRad, Watford, UK).

### 4.8. Statistical Analysis

Data were normally distributed and therefore analysed using Student’s *t*-test to compare between two samples, or one-way ANOVA with post hoc Tukey’s test to compare between multiple samples using GraphPad Prism version 6. The details of all statistical analyses are shown in Supplementary Table S1.

## Data Availability

Data will be made available upon request.

## Supplementary Materials

The supporting information can be downloaded at: https://www.mdpi.com/article/10.3390/ijms23010554/s1.
