# Supplementary material for: The microRNA-455 Null Mouse Has Memory Deficit and Increased Anxiety, Targeting Key Genes Involved in Alzheimer’s Disease
Source: Int J Mol Sci. 2022 Jan 5;23(1):554. doi: 10.3390/ijms23010554 (PMC8745123; doi:10.3390/ijms23010554)
Supplement: Supplementary file 1 [file ijms-23-00554-s001.zip › Table S1.pdf]

Table S1: Statistical analyses

Experiment	Dataset 1 (mean+/- SEM)	Dataset 2 (mean+/- SEM)	p-value
Weight of 14 month old mice, wild-type vs null	38.07±1.950	45.59±2.464	0.025
Level of miR-455-3- across age 3 weeks vs 1 year	$2.94 \times 10^{-6} \pm 5.57 \times 10^{-7}$	$1.04 \times 10^{-6} \pm 3.30 \times 10^{-7}$	0.04
Novel object recognition, wild-type vs null	0.400±0.094	-0.0163±0.074	0.0038
Open field test, time in centre, wild-type vs null	8.450±1.613	4.814±1.087	0.09
Open field test, total distance travelled	77.25±8.081	74.03±5.795	NS
Luciferase activity, BACE1 UTR ± miR-455-3p	1.019±0.048	0.8023±0.030	0.008
Luciferase activity, BACE1 UTR mutant ± miR-455-3p	1.035±0.063	0.987±0.096	NS
Luciferase activity, TAU UTR ± miR-455-3p	20.901±0.017	1.711±0.055	0.0006
Luciferase activity, TAU UTR mutant ± miR-455-3p	2.175±0.055	2.294±0.246	NS
APP, SH-SY5Y cells, qRT-PCR, ± miR-455-3p	$1.02 \times 10^{-4} \pm 1.68 \times 10^{-5}$	$6.40 \times 10^{-5} \pm 5.93 \times 10^{-6}$	NS
BACE1, SH-SY5Y cells, qRT-PCR, ± miR-455-3p	$3.31 \times 10^{-5} \pm 2.94 \times 10^{-6}$	$2.30 \times 10^{-5} \pm 3.03 \times 10^{-6}$	NS (0.09)
TAU, SH-SY5Y cells. qRT-PCR, ± miR-455-3p	$4.31 \times 10^{-5} \pm 6.67 \times 10^{-6}$	$2.72 \times 10^{-5} \pm 2.82 \times 10^{-6}$	NS (0.09)
APP, hippocampus, qRT-PCR, WT vs null	$9.66 \times 10^{-4} \pm 6.20 \times 10^{-5}$	$8.75 \times 10^{-4} \pm 4.53 \times 10^{-5}$	NS
BACE1, hippocampus, qRT-PCR, WT vs null	$2.94 \times 10^{-5} \pm 2.50 \times 10^{-6}$	$3.19 \times 10^{-5} \pm 2.16 \times 10^{-6}$	NS
TAU, hippocampus, qRT-PCR, WT vs null	$1.87 \times 10^{-4} \pm 8.01 \times 10^{-5}$	$2.26 \times 10^{-4} \pm 1.37 \times 10^{-5}$	P=0.046
APP, hippocampus, western blot, WT vs null	1.087±0.176	2.544±0.049	0.0013
BACE1, hippocampus, western blot, WT vs null	0.694±0.105	1.016±0.039	0.0447
TAU, hippocampus, western blot, WT vs null	0.364±0.147	0.607±0.173	NS
