# Supplementary figures and images for: The microRNA-455 Null Mouse Has Memory Deficit and Increased Anxiety, Targeting Key Genes Involved in Alzheimer’s Disease
Source: Int J Mol Sci. 2022 Jan 5;23(1):554. doi: 10.3390/ijms23010554 (PMC8745123; doi:10.3390/ijms23010554)

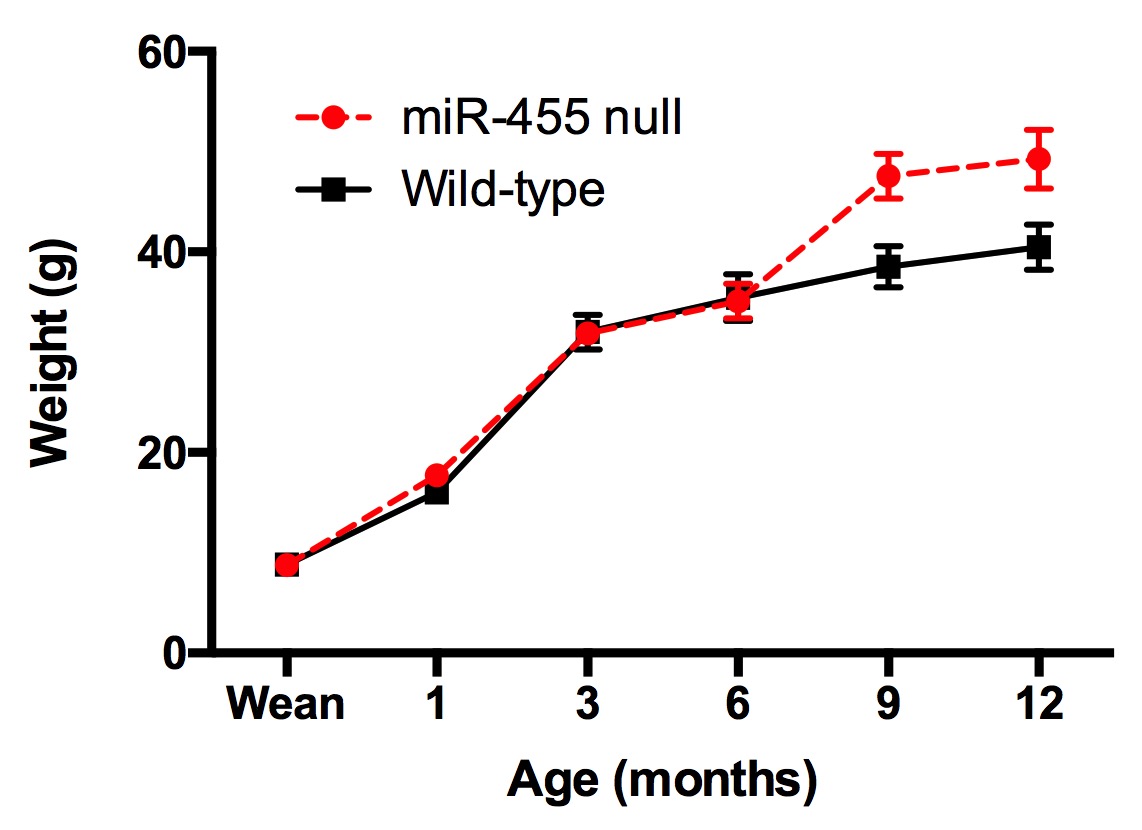

Supplement: Supplementary file 1 [file ijms-23-00554-s001.zip › Figure S1.jpg]

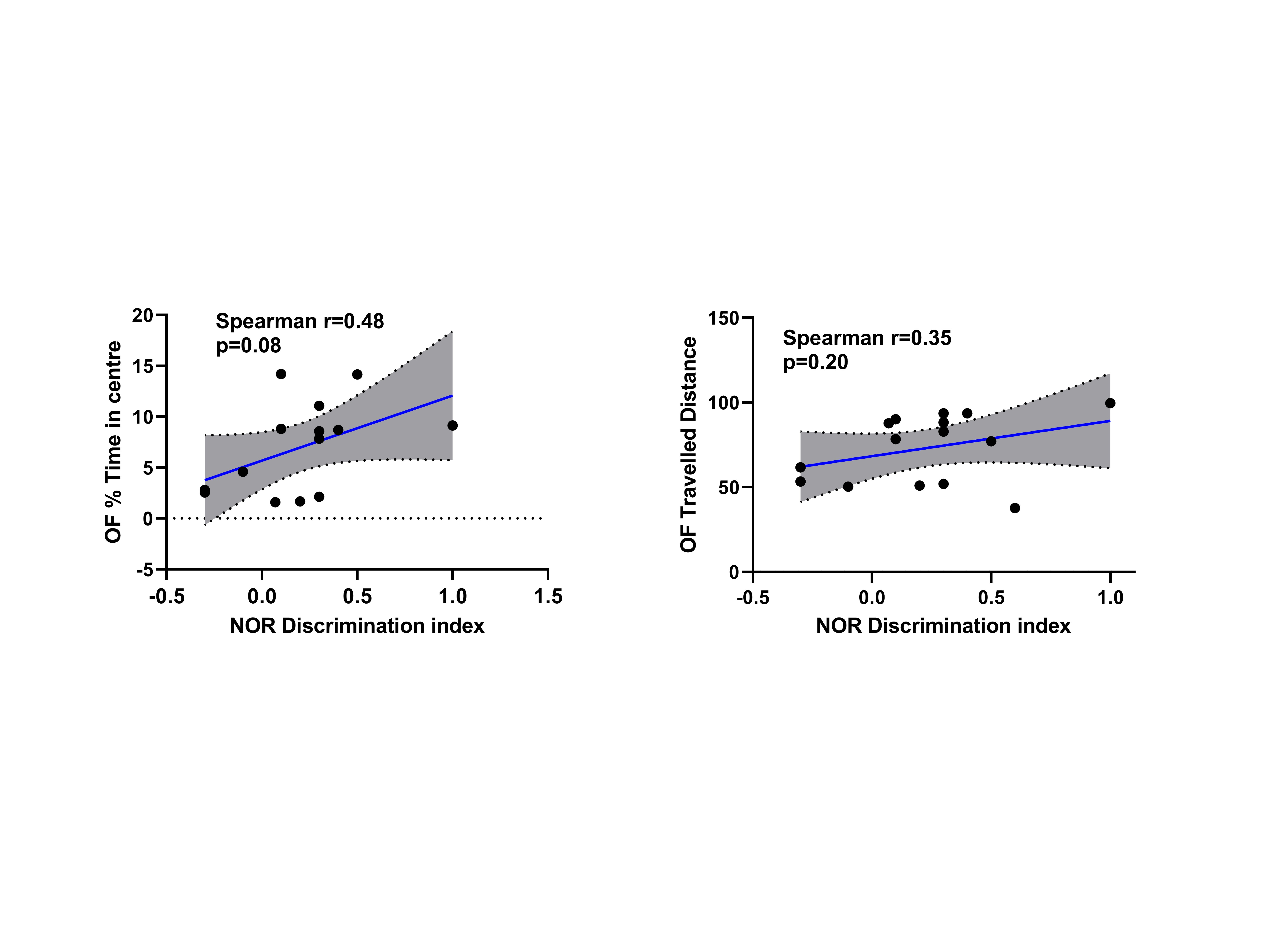

Supplement: Supplementary file 1 [file ijms-23-00554-s001.zip › Figure S2.JPG]
